# Comparison of comfort and complications of Implantable Venous Access Port (IVAP) with ultrasound guided Internal Jugular Vein (IJV) and Axillary Vein/Subclavian Vein (AxV/SCV) puncture in breast cancer patients: a randomized controlled study

**DOI:** 10.1186/s12885-022-09228-6

**Published:** 2022-03-05

**Authors:** Yan Bo Chen, Hao Shi Bao, Ting Ting Hu, Zhou He, Biaolin Wen, Feng Tao Liu, Feng Xi Su, He Ran Deng, Jian Nan Wu

**Affiliations:** 1 Guangdong Provincial Key Laboratory of Malignant Tumor, Epigenetics and Gene Regulation, Sun Yat-sen Memorial Hospital, Sun Yat-sen University, Guangzhou, People’s Republic of China; 2 Department of Orthopedics, Sun Yat-sen Memorial Hospital, Sun Yat-sen University, Guangzhou, People’s Republic of China; 3 S’ CLINIC, Guangzhou, People’s Republic of China; 4 Breast Tumor Center, Sun Yat-sen Memorial Hospital, Sun Yat-sen University, Guangzhou, People’s Republic of China

**Keywords:** Implantable venous access port (IVAP), Axillary vein/subclavian vein (AxV/SCV), Internal jugular vein (IJV), Comfort, Complications

## Abstract

**Background:**

Axillary vein/subclavian vein (AxV/SCV) and Internal jugular vein (IJV) are commonly used for implantable venous access port (IVAP) implantation in breast cancer patients for chemotherapy. Previous research focused on comparison of complications while patient comfort was ignored. This study aims to compare patient comfort, surgery duration and complications of IVAP implantation between IJV and AxV/SCV approaches.

**Methods:**

Two hundred forty-eight breast cancer patients were enrolled in this randomized controlled study from August 2020 to June 2021. Patients scheduled to undergo IVAP implantation were randomly and equally assigned to receive central venous catheters with either AxV /SCV or IJV approaches. All patients received comfort assessment using a comfort scale table at day 1, day 2 and day 7 after implantation. Patient comfort, procedure time of operation as well as early complications were compared.

**Results:**

Patient comfort was significantly better in the AxV/SCV group than that of IJV group in day 1 (*P* < 0.001), day 2 (*P* < 0.001) and day 7(*P* = 0.023). Procedure duration in AxV/SCV group was slightly but significantly shorter than IJV group (27.14 ± 3.29 mins vs 28.92 ± 2.54 mins, *P* < 0.001). More early complications occurred in AxV/SCV group than IJV group (11/124 vs 2/124, *P* = 0.019). No difference of complications of artery puncture, pneumothorax or subcutaneous hematoma between these two groups but significantly more catheter misplacement in AxV/SCV group than IJV group (6/124 vs 0/124, *P* = 0.029). Absolutely total risk of complications was rather low in both groups (8.87% in AxV/SCV group and 1.61% in IJV group).

**Conclusions:**

Our study indicates that patients with AxV/SCV puncture have higher comfort levels than IJV puncture. AxV/SCV puncture has shorter procedure duration but higher risk of early complications, especially catheter misplacement. Both these two approaches have rather low risk of complications. Consequently, our study provides an alternative choice for breast cancer patients to reach better comfort.

## Background

Breast cancer is the most common malignant tumor and leads to the most cancer deaths in females [1, 2]. Systemic intravenous chemotherapy has been recommended to many of invasive breast cancer patients to reduce the recurrence risk and improve patient prognosis, with the exception of those patients who are at low recurrence risk [3, 4]. As we know, it is dangerous to administer chemotherapy through peripheral veins because there are severe side effects such as chemotherapy drugs extravasation, unacceptable pain and psychological trauma [5, 6]. Hence, long-term CVC infusion ports are usually recommended for breast cancer patients for their convenience and safety during chemotherapy [7].

The most commonly used puncture sites of central venous catheters are subclavian veins (SCVs) and internal jugular veins (IJVs) [8]. In fact, axillary vein (AxV), the direct continuation of the SCV, has also been considered as an important alternative option of CVC puncture [9–11]. Ultrasound guidance is the standard technique during CVC insertion via both IJV and SCV [12, 13]. Compared with anatomical landmark guidance, procedure guided by ultrasound has been proved to improve patient safety and comfort [14].

Previous studies usually focused on the comparison of complications and safety between puncture via SCV or AxV and IJV [11, 15, 16]. However, to our knowledge, there was no study focusing on the comparison of patient comfort. Consequently, this work aims to compare patient comfort after IJV or AxV /SCV puncture with the help of ultrasound guidance. At the same time, we also compare procedure duration and complications between these two groups. The protocol of this study has been peer reviewed and published [17].

## Methods

### Patient selection

Inclusion criteria in this study were: (I) age 18–70 years old; (II) histologically confirmed diagnosis of invasive breast cancer; (III) with indication of chemotherapy according to the latest NCCN guideline; (IV) with the ability to understand our comfort scale questionnaire and comply with the study protocol.

Cases with one of these following situations or more were excluded: (I) confirmed bilateral breast cancer; (II) confirmed distant metastasis; (III) have coagulation diseases history such as deep venous thrombosis (DVT).

### Study design

**Fig. 1:**
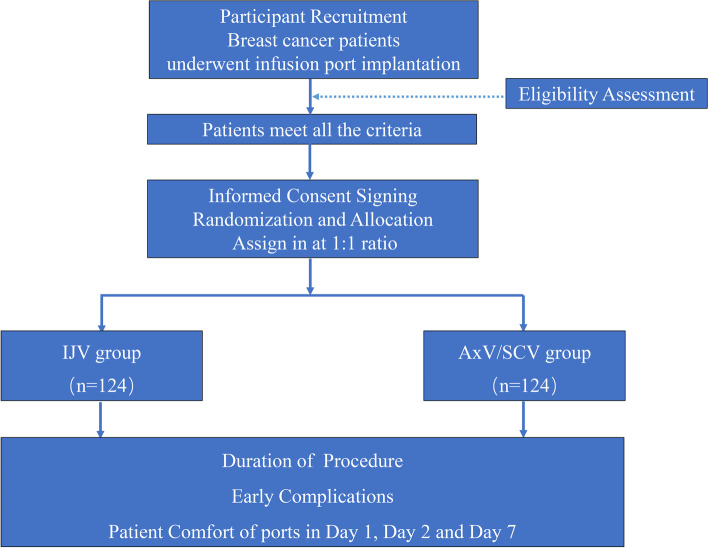
Flow diagram of the progress through the study

This was a prospective single center randomized controlled study. Breast cancer patients received port implantation from August 2020 to June 2021 were randomly assigned to AxV /SCV group or IJV group by simple randomization in a 1:1 ratio with computerized system for random numbers generation. Two designated doctors (Dr. Bao and Dr. Deng) with expertise in IVAP implantation performed the procedures for all patients. Patients enrolled completed all preoperative examinations before port implantation, including coagulation screening, blood routine examination, electrocardiograph, and puncture vascular ultrasound. After implantation surgery, all patients received comfort assessment at day 1, day 2 and day 7 with a comfort scale table. The study flowchart is shown in Fig. 1.

### Implantation procedure

**Fig. 2:**
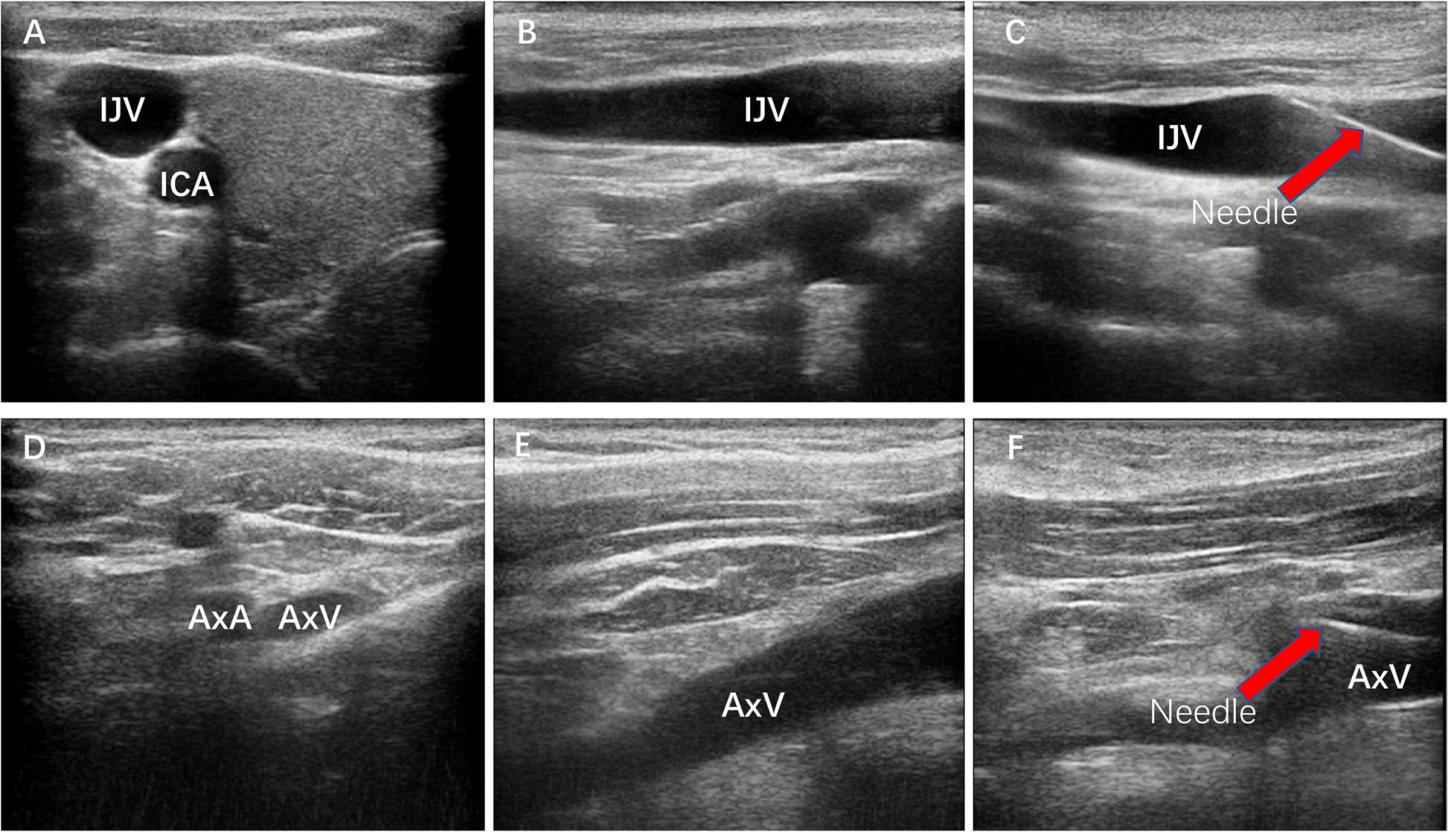
Center venous catheter puncture with ultrasound guidance. **A**-**C** Center venous catheter puncture through IJV with ultrasound guidance. **D**-**F** Center venous catheter puncture through AxV/SCV with ultrasound guidance. ICV: Internal carotid artery. IJV: Internal jugular vein. AxA: Axillary artery. AxV: Axillary vein. SCV: Subclavian vein

All the procedures were performed strictly following the aseptic principles in the out-patient operation room. Vital signs were monitored using electrocardiogram monitor, noninvasive blood pressure cuff and pulse oximetry continuously. For IJV group, patients were positioned with the head turning to the left in a neutral supine position while for AxV/SCV group, patients were positioned in a neutral supine position. Lidocaine (2%) was used for local anesthesia and no sedatives were used during procedure. One of the three designated brands of infusion ports, BARD (Bard Limited, Salt Lake City, USA), B Braun (B.Braun Medical, Chasseneuil-du-Poitou, France) and Medcomp (Medical Components, Inc., Harleysville, USA) was used for patients according to the surgeon’s preference. Guide wire and catheter insertion were performed with the ultrasound guidance (MYLAB 30CV, Esaote Medical Technology Co. LTD, China) (Fig. 2).

**Fig. 3:**
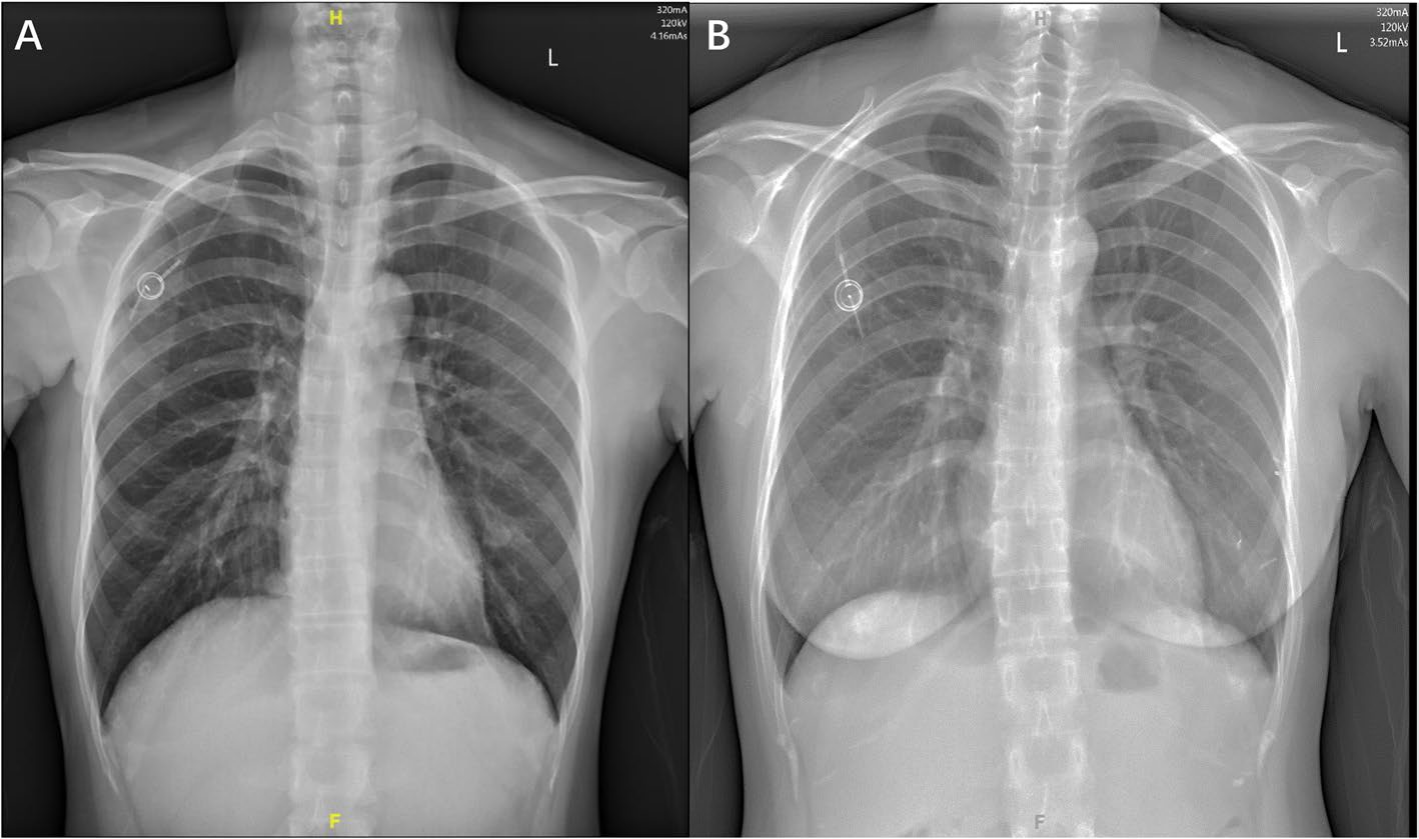
Chest X-rays of patients right after infusion ports implantation. **A** Chest X-ray of patient with CVC puncture through IJV. **B** Chest X-ray of patient with CVC puncture through AxV/SCV. CVC: Center Venous Catheter. IJV: Internal jugular vein. AxV/SCV: Axillary/Subclavian vein

For patients in IJV group, we inserted the guide wire and then catheter was replaced through right IJV. For AxV/SCV group patients, we inserted the guide wire and then replaced with catheter through AxV or right SCV. All patients took a chest X-ray right after the procedure (Fig. 3) and then we checked the early complications.

### Outcome measures

The primary outcome measure of this study is patient comfort at Day 7, 1 week after CVC puncture. Secondary outcome measures are patient comfort at Day 1, just after CVC puncture surgery and Day 2, 1 day after CVC puncture. The comfort scale in our study (Table 1) is a visual analogic scale, which has been applied and proved to be a simple and effective method for patients’ comfort or satisfaction assessment [18, 19].

**Table 1:** Patient comfort scale table

Grade	Discomfort Scale
Grade 0	Without any discomfort
Grade 1	Extremely mild discomfort
Grade 2	A little discomfort
Grade 3	Some discomfort
Grade 4	Rather uncomfortable
Grade 5	Extreme discomfort

Other secondary outcome measures of our study are as follows: (I) duration of procedure (minutes), defined as the time between the initial cutaneous sterilization and dressing placement; (II) early complications rate. Early complication is defined as the period of time between intraoperative implantation and the first use of catheter, which includes wire advancement difficulties, inadvertent artery puncture, catheter misplacement, pneumothorax or subcutaneous hematoma. All these complications are defined as described in the protocol [17].

### Statistical analysis

All the statistical analysis were performed with GraphPad Prism version 8.0 for Windows (GraphPad Software Inc., San Diego, California, USA) and IBM SPSS Statistics for Windows, version 27 (IBMCorp., Armonk, NY., USA). Summary statistics for quantitative variables with normal distribution were expressed with means and standard deviations. Unordered categorical variables were summarized with percentages or ratios. Differences in means of continuous variables were compared with Student’s t-test (two groups). Discontinuous variables were compared using Wilcoxon rank test, and differences in proportions were tested by Chi-Square test. *P* < 0.05 is considered statistically significant.

### Ethics and informed consents

The study was reviewed and approved by the medical ethics committee of Sun Yat-Sen Memorial Hospital, Sun Yat-Sen University (No.2020-KY-053) and followed the provisions of Helsinki Declaration. This study has also been registered on 26/07/2020 in Chinese Clinical Trial Registry (ChiCTR, www.chictr.org.cn) and Chinese Ethics Committee of Registering Clinical Trials (No.ChiCTR2000034986). All patients provided written informed consent to participate in this study and additional consent before surgical procedures. All methods were carried out in accordance with relevant guidelines and regulations.

## Results

### Baseline characteristics of patients

Total of 248 patients were included in this study and each group had 124 patients. Three patients in IJV group and one patient in AxV/SCV group were lost to follow up at day 2 and day 7.

No significant differences of age, BMI, marriage status and history of diabetes between IJV group and AxV/SCV group at baseline. Tumor side, chemotherapy, breast and lymph node surgery were also without significant difference. Detailed data was showed in Table 2.

**Table 2:** Baseline and Demographic Data of IJV group and AxV/SCV group

	Overall population *n* = 248	IJV group *n* = 124	AxV/SCV group *n* = 124	*P*
Age (years)	46.21 ± 9.80	47.38 ± 10.23	45.05 ± 9.24	0.061
BMI (kg/m^2^)				
< 18	9 (3.6%)	5 (4.0%)	4 (3.2%)	0.080
18–24	174 (70.2%)	79 (63.7%)	95 (76.6%)	
> 24	65 (26.2)	40 (32.3%)	25 (20.2%)	
Marriage status				
Married	221 (89.1%)	109 (87.9%)	112 (90.3%)	0.651
Unmarried	16 (6.5%)	8 (6.5%)	8 (6.5%)	
Divorced or widowed	11 (4.4%)	7 (5.6%)	4 (3.2%)	
Tumor side				
Left	128 (51.6%)	59 (47.6%)	69 (55.6%)	0.253
Right	120 (48.4%)	65 (52.4%)	55 (44.4%)	
History of Diabetes				
Yes	7 (2.8%)	3 (2.4%)	4 (3.2%)	1.000
No	241 (97.2%)	121 (97.6%)	120 (96.8%)	
Chemotherapy				
Adjuvant	207 (83.5%)	105 (%)	102 (%)	0.733
Neoadjuvant	41 (16.5%)	19 (%)	22 (%)	
Breast Surgery				
BCS	164 (66.1%)	79 (63.7%)	85 (68.5%)	0.608
MRM	69 (27.8%)	38 (30.6%)	31 (25.0%)	
BRS	15 (6.0%)	7 (5.6%)	8 (6.5%)	
Lymph Node Surgery				
ALND	108 (43.5%)	56 (45.2%)	52 (41.9%)	0.701
SLNB	140 (56.5%)	68 (54.8%)	72 (58.1%)	

1. Continuous variables were expressed as means and standard deviations (mean ± standard deviation) while categorical variables were summarized by number and percentages: n (%). Differences in means for continuous variables were compared using Student’s t-test and differences in proportions were tested by Chi-Square test

2.*Abbreviation*: *BMI* Body Mass Index, *BCS* Breast Conserving Surgery, *MRM* Modified Radical Mastectomy, *BRS* Breast Reconstruction Surgery, *ALND* Axillary Lymph Node Dissection, *SLNB* Sentinel Lymph Node Biopsy

### Patient comfort of the two groups of patients

**Fig. 4:**
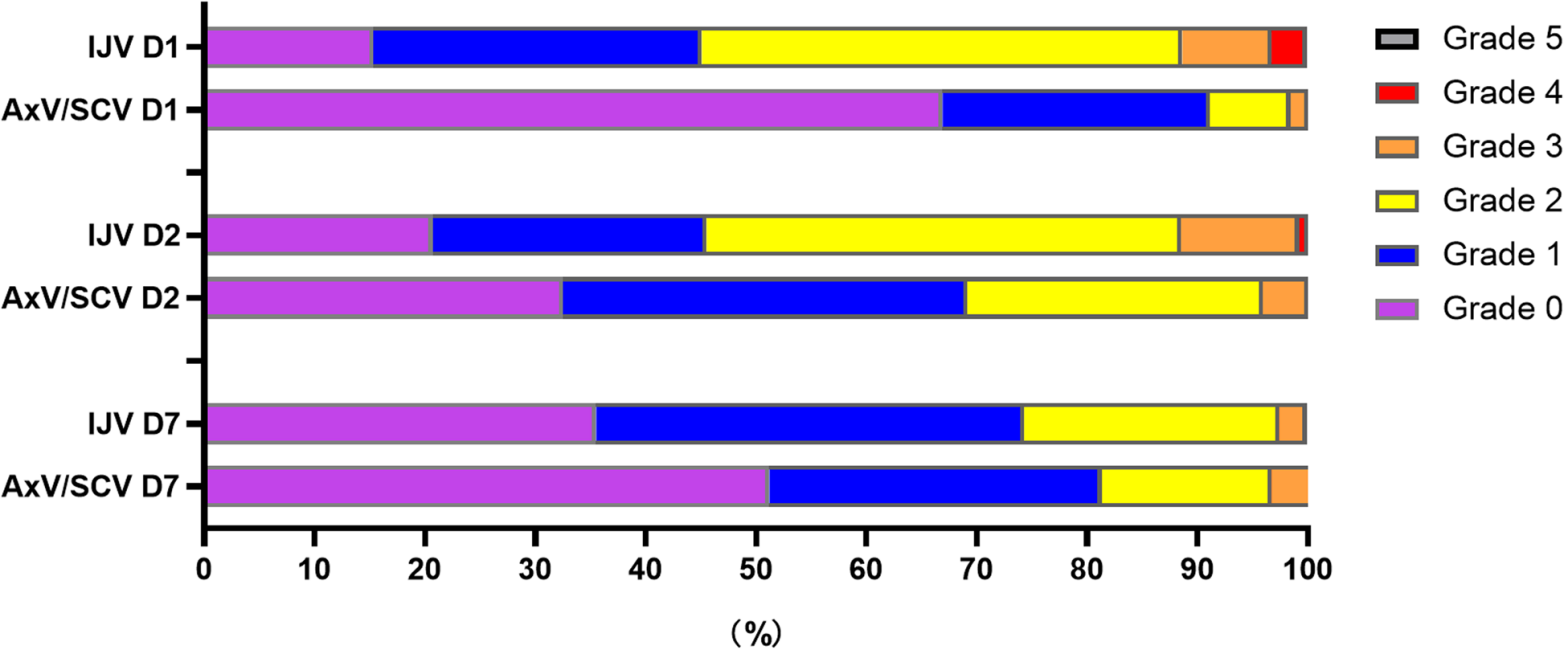
Patient comfort of AxV/SCV group and IJV group on day 1, day 2 and day 7

Patient comfort of AxV/SCV group was significantly better than that of IJV group on day 1 (*P* < 0.001), day 2 (*P* < 0.001) and day 7 (*P* = 0.023). On day 1, most patients had grade 1 (37/124, 29.8%) or grade 2 (54/124, 43.5%) in IJV group while most patients had grade 0 (83/124, 66.9%) or grade 1 (30/124, 24.2%) in AxV/SCV group. On day 2, about half the patients had grade 2 (52/121, 43.0%) and grade 3 (13/121, 10.7%) in IJV group while only about 30% of the patients of grade 2 (33/123, 26.8%) and grade 3 (5/123, 4.1%) in AxV/SCV group. On day 7, 51.2% cases had grade 0 and 30.1% had grade1 in AxV/SCV group while 35.5% cases had grade 0 and 38.8% cases had grade 1 in IJV group. Only four patients had grade 4 on day 1 and one case on day 2 in IJV group and no patients had grade 4 in AxV/SCV group. No patients had grade 5 on both groups. Detailed data was showed in Table 3 and Fig. 4.

**Table 3:** Patient comfort of IJV group and AxV/SCV group

Patient Comfort (Grade)	0	1	2	3	4	5	*P*
**D1**							
IJV group (*n* = 124)	19 (15.3%)	37 (29.8%)	54 (43.5%)	10 (8.1%)	4 (3.2%)	0	< 0.001*
AxV/SCV group (*n* = 124)	83 (66.9%)	30 (24.2%)	9 (7.3%)	2 (1.6%)	0	0	
**D2**^**a**^							
IJV group (*n* = 121)	25 (20.7%)	30 (24.8%)	52 (43.0%)	13 (10.7%)	1 (0.8%)	0	< 0.001*
AxV/SCV group (*n* = 123)	40 (32.5%)	45 (36.6%)	33 (26.8%)	5 (4.1%)	0	0	
**D7**^**a**^							
IJV group (*n* = 121)	43 (35.5%)	47 (38.8%)	28 (23.1%)	3 (2.5%)	0	0	0.023*
AxV/SCV group (*n* = 123)	63 (51.2%)	37 (30.1%)	19 (15.4%)	4 (3.3%)	0	0	

1. Patient comfort between IJV group and AxV/SCV group on D1, D2, D7 were compared by Wilcoxon rank test. *Data significant at *P* < 0.05

2. ^a^3 patients from IJV group and 1 patient from AxV/SCV group were lost to follow up on day 2 and day 7, so only total of 244 patients were included in analysis

### Procedure duration and complications of the two groups of patients

**Fig. 5:**
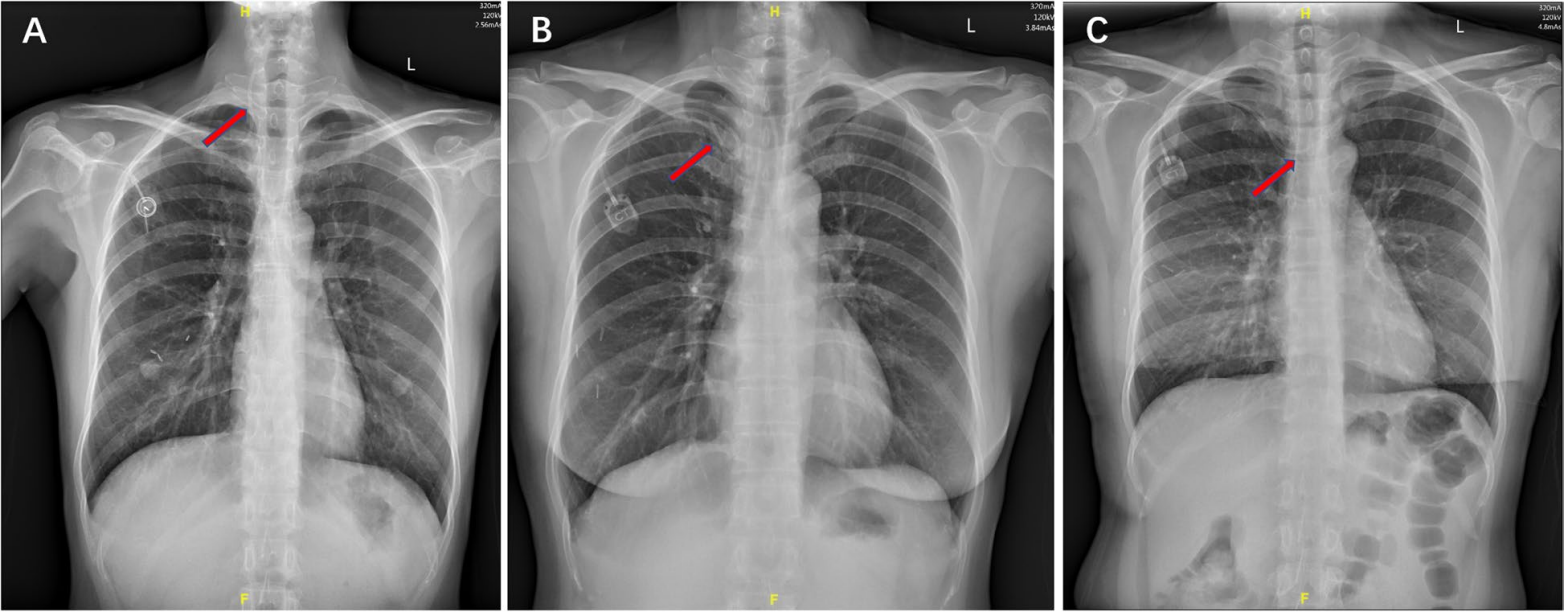
Catheter misplacement in the AxV/SCV group patients. The red arrow indicates the misplaced catheters. **A** Catheter misplacement at right internal jugular vein. **B** Catheter misplacement at right subclavian vein. **C** Catheter misplacement at left brachiocephalic vein

Procedure duration was 27.14 ± 3.29 mins in AxV/SCV group, which was significantly shorter than that 28.92 ± 2.54 mins of IJV group (*P* < 0.001). There were totally 11 cases with early complications in AxV/SCV group and 2 cases in IJV group (8.87% vs 1.61%, *P* = 0.019). Among 11 cases with complications in AxV/SCV group, 6 cases had catheter misplacement, 2 cases had subcutaneous hematoma and 3 cases had artery puncture. Only 1 case had subcutaneous hematoma and 1 case had artery puncture in the IJV group. No significant difference of artery puncture or subcutaneous hematoma occurred in these two groups and no severe complications such as pneumothorax or hemothorax were observed. However, there was much more catheter misplacement in the AxV/SCV group than the IJV group (4.84% vs 0%, *P* = 0.029). Detail was showed in Table 4. Among these 6 cases of catheter misplacement, the catheter tips were located at right internal jugular vein in 2 cases, at right subclavian vein in 2 cases and at left brachiocephalic vein in 2 cases (Fig. 5).

**Table 4:** Procedure Duration and Complications of IJV group and AxV/SCV group

	Overall population *n* = 248	IJV group *n* = 124	AxV/SCV group *n* = 124	*P*
Procedure duration (min)	28.03 ± 3.06	28.92 ± 2.54	27.14 ± 3.29	< 0.001*
Early complications (total)	13 (5.24%)	2 (1.61%)	11 (8.87%)	0.019*
Inadvertent artery puncture	4 (1.61%)	1 (0.81%)	3 (2.42%)	0.622
Pneumothorax	0	0	0	–
Subcutaneous hematoma	3 (1.21%)	1 (0.81%)	2 (1.61%)	1.000
Catheter misplacement	6 (2.42%)	0	6 (4.84%)	0.029*

1. Procedure duration and complications were compared between IJV group and AxV/SCV group. Differences in means were test by t-test, while differences in proportions were tested by Chi-Square test. *Data significant at *P* < 0.05

## Discussion

As far as we know, this is the first study to compare patients’ comfort as primary outcome measure between IJV and AxV/SCV puncture. In this study, we found that patients underwent IVAP implantation through AxV/SCV had better comfort than those through IJV on day 1, day 2 and day 7 after puncture. At the same time, the duration time of AxV/SCV puncture was slightly shorter than IJV puncture. Although the risk of complications was higher in patients with AxV/SCV puncture, the totally risk of complications was rather low in both AxV/SCV group and IJV group with ultrasound guidance. In a word, our study provides an alternative better choice of IVAP implantation for breast cancer patients to get better comfort.

As previously described, over the years, an enormous amount of research comparing AxV/SCV and IJV has focused on the complications and safety of these two insertion approaches. However, patient satisfaction and comfort were only mentioned in only a few studies [19, 20]. In this prospective randomized controlled study, we compared patients’ comfort of IVAP implantation as primary outcome measure, and this is the first study to put patient comfort in the forefront. Our study indicated that patients in AxV/SCV group had significantly higher comfort level than those in IJV group. Many IJV group patients were afraid to turn their neck because they felt a foreign body sensation in their neck and this uncomfortable feeling may last for several days after the CVC insertion. Consequently, our study can provide an alternative choice for patients to reach better comfort level.

Our study also compared the procedure duration of CVC puncture between these two approaches and we found that it was significantly shorter in AxV/SCV group than IJV group, although the difference of less than 2 mins was negligible. However, past studies showed that the access time for the first attempt and the average whole procedure time was significantly longer in AxV/SCV group than IJV group [21, 22]. This difference may be explained by the following reasons. Firstly, we used ultrasound guidance and this has been proved to significantly reduce the procedure duration of CVC puncture [23]. Secondly, there are two incisions during procedure in IJV group patients while only one incision in AxV/SCV group. At the same time, subcutaneous tunneling construction is not required during IVAP implantation in AxV/SCV group. Consequently, with the help of ultrasound guidance, the procedure time is slightly shorter in AxV/SCV group than IJV group, and this may be one of the advantages of AxV/SCV puncture.

Comparison of complication incidence between AxV/SCV and IJV approach still remains controversial. Some studies demonstrated higher complications risk such as pneumothorax or symptomatic venous thrombosis in SCV puncture than IJV puncture [22, 24, 25]. However, other research indicated no difference of complication risk between these two different insertion ways [11, 16, 26]. A multicenter randomize trial with 3471 catheters indicated that SCV catheterization had a lower risk of symptomatic thrombosis and bloodstream infection than femoral vein or IJV catheterization but a higher risk of pneumothorax [27]. Another study demonstrated no significant difference of total complication rate between SCV and IJV but there seemed to be more catheter misplacements in SCV catheterization [21], which is consistent with our study. In our study, we found that there are much more catheter misplacements in the AxV/SCV group. In order to decrease the risk of catheter misplacements after IVAP implantation, we can use ultrasound to detect whether the catheter is at the right internal jugular vein or not immediately after the catheter placement. If the catheter is located at the right internal jugular vein, we can withdraw the catheter and replace it again to make sure that the catheter is not at internal jugular vein. However, if the catheter is misplaced at right subclavian vein or left brachiocephalic vein, the ultrasound cannot observe it and we can only observe this after X-ray examination, so these cases should receive operation of catheter replacement again. Generally, in this study, we found that complication rate patients developed was rather low in both IJV group and AxV/SCV group with ultrasound guidance and there were no severe complications cases such as pneumothorax or hemothorax. Hence, our study further confirms the benefits from ultrasound guidance for its safety and convenience and we strongly recommend ultrasound guidance in IVAP implantation. However, further studies to reduce the risk of catheter misplacement during AxV/SCV puncture are of great significance in the future.

There are still some limitations in this study. Firstly, our study was not blinded to patients and may influence patient comfort to some extent. Also, we did not compare late complications in our study. Lastly, this was a single center research and the sample size was not large enough; hence, it is necessary to decrease the risk of catheter misplacement in the future, and to perform a prospective multicenter survey to compare patient comfort between AxV/SCV puncture and IJV puncture with a larger sample size.

## Data Availability

All data generated or analyzed during this study are included in this published article.

## Abbreviations

CVC: Central Venous Catheters
IVAP: Implantable venous access port
IJV: Internal Jugular Vein
SCV: Subclavian Vein
AxV: Axillary Vein
DVT: Deep venous thrombosis
